# Risk Factor Impact on African Swine Fever Transmission in Different Extensive Pig Production Settings in Serbia

**DOI:** 10.3390/v15061232

**Published:** 2023-05-24

**Authors:** Jan Plut, Melita Hajdinjak, Jasna Prodanov-Radulović, Siniša Grubač, Biljana Djurdjević, Marina Štukelj

**Affiliations:** 1 Clinic for Ruminants and Pigs, Clinic for Reproduction and Large Animals, Veterinary Faculty, University of Ljubljana, 1000 Ljubljana, Slovenia; marina.stukelj@vf.uni-lj.si; 2 Laboratory of Applied Mathematics and Statistics, Faculty of Electrical Engineering, University of Ljubljana, 1000 Ljubljana, Slovenia; melita.hajdinjak@fe.uni-lj.si; 3 Scientific Veterinary Institute Novi Sad, 21000 Novi Sad, Serbia; jasna@niv.ns.ac.rs (J.P.-R.); grubac@niv.ns.ac.rs (S.G.); biljana@niv.ns.ac.rs (B.D.)

**Keywords:** African swine fever, backyards, biosecurity, Serbia, risk factors

## Abstract

The first case of ASF in a domestic pig population in Serbia was confirmed in 2019 in a backyard population. Today, outbreaks in wild boar and, more importantly, in domestic pigs are still occurring, although the government measures for ASF prevention are in place. The aim of this study was to determine critical risk factors and identify the possible reasons for ASF introduction into different extensive pig farms. The study was conducted on 26 extensive pig farms with confirmed ASF outbreaks, with data collected from beginning of 2020 to the end of 2022. Collected epidemiological data were divided into 21 main categories. After identifying specific values of variables as critical for ASF transmission, we identified nine important ASF transmission indicators as those variables for which at least 2/3 of the observed farms reported values critical for ASF transmission. Among them were type of holding, distance to hunting ground, farm/yard fencing, and home slaughtering; however, the hunting activity of pig holders, swill feeding, and feeding with mowed green mass were not included. We represented the data in the form of contingency tables to study associations between pairs of variables using Fisher’s exact test. All pairs of variables in the group including type of holding, farm/yard fencing, domestic pig–wild boar contact, and hunting activity were significantly related; hunting activity of pig holders, holding pigs in backyards, unfenced yards, and domestic pig–wild boar contact were observed on the same farms. Free-range pig farming led to observed domestic pig–wild boar contact on all farms. The identified critical risk factors need to be strictly addressed to prevent the further spread of ASF to extensive farms and backyards in Serbia and elsewhere.

## 1. Introduction

African swine fever (ASF) is a viral disease caused by an *Asfavirus* that affects domestic pigs and wild boars [1,2] It can cause a high mortality rate in infected animals, and because there is no treatment or vaccination, prevention and control of ASF currently rely on preventive and biosecurity measures [3,4]. ASF is an infectious, contagious disease that can be transmitted through direct and/or indirect contact with infected animals, contaminated clothing and equipment, and the consumption of contaminated meat products [4,5,6]. Since the introduction of African swine fever virus (ASFV) to Georgia in 2007 [7], the disease has gradually spread throughout the European continent [8]. According to available data, most ASF reports in Europe were recorded in wild boars, suggesting that the wild boar population is currently the predominant host of ASF [9]. However, the ASF-infected wild boar population is known to pose a serious threat to the domestic pig industry [10]. More importantly, the occurrence of ASF in an affected country results in severe constraints for swine producers [11,12]. Therefore, preventive measures, as well as surveillance activities that record contact between domestic pig and wild boar populations, are of utmost importance, not only for disease control but also to detect new cases as soon as possible [13].

The first case of ASF in a domestic pig herd in Serbia was confirmed in 2019 in backyards in a village in the central region of the country [14]. Shortly afterward, in January 2020, the first case was detected in a wild boar population that occurs predominantly in open hunting grounds in counties close to the country’s borders with Romania and Bulgaria [15]. Numerous outbreaks were later reported in feral pigs near the border area in southeastern Serbia from 2020 to the end of 2022 [16]. It was reported that in two counties bordering the country, ASF outbreaks in domestic pigs frequently overlapped with the occurrence of ASF in wild boar populations, suggesting that regional prevalence in wild boars is an important risk factor for domestic pig populations [17].

As of 2023, ASF has been present in Serbia for almost four years, and the authorities are taking numerous preventive measures to control its spread [11,18]. The Serbian Government has taken several important ASF control measures, including the culling of infected animals (stamping out) and strict restrictions on the movement of pigs and pig products within affected areas and from these areas to uninfected areas. For wild boars, the main measures include passive and active surveillance of hunting areas throughout the national territory. These preventive measures have also included the reduction in wild boar populations to a biological minimum in high-risk areas, which contributed, to some degree, to the control of ASF in the wild boar population. ASF is a notifiable disease, which means that it must be reported to the relevant authorities when it is detected [18,19,20]. The authorities have also introduced a compensation scheme for farmers, and the government provides 100% compensation to farmers for stamping out domestic pigs in the event of an ASF outbreak and for preventive depopulation of the surrounding area [21].

In southeastern Europe, the existence of a highly variable domestic pig husbandry industry was previously reported in [22]. The most important difference between the EU member countries is the structure of pig production sector. In this region, there are a large number of smallholdings, and semi-free-range and free-range domestic pigs are reported in some countries [12,17]. According to the EFSA report [12], in most non-EU countries of the Western Balkans, a significant percentage of domestic pig population occurs in backyard production systems.

Pig farming is an important sector in Serbia, with many different types of farms producing pork for both the domestic market and for own consumption. However, the characteristics of pig farming vary greatly depending on the region of the country. Intensive commercial systems exist mainly in the north of the country (Vojvodina province), where pigs are kept in indoor facilities and biosecurity measures are most stringent [15]. At the same time, however, in the vicinity of the intensive production units, in the surrounding villages, there are a considerable number of different types of family farms (smallholdings), which are often more traditional and semi-extensive or extensive types of pig production. In Serbia, the classification of the pig sector includes four main types of pig production farms: commercial pig farms (farrow-to-finish or farrow-to-piglet farms or pure fattening farms and artificial insemination centers) have the highest level of biosecurity. In terms of the biosecurity level and the number of animals, the next category is Type A family farms: these are family farms with more than 10 pigs, including breeding categories. At Type B family farms, which also have more than 10 pigs, biosecurity measures are less stringently implemented. The next category is backyard farms, which usually have fewer than 10 animals; in this type of production, biosecurity measures are low or often inefficient. Finally, extensive semi-enclosed and free-range production includes animals kept in semi-fenced or unenclosed areas without the use of biosecurity measures [17]. It should be emphasized that, in Serbia, biosecurity measures for pig production are not officially required by law and are given only in the form of general recommendations. The recent government instruction on ASF only requires that commercial (intensive production) and type A family farms have an official written and implemented biosecurity plan specific to the farm conditions and production orientation. However, it does not provide any details on what the biosecurity plan must contain [15]. It is well known that preventing ASF transmission on smallholder farms and backyard systems is key to sustainable ASF control [4,17,23]. Although the factors and risks for ASF transmission are known and frequently discussed, the situation regarding these factors in different livestock production environments in Serbia is largely undetermined and underassessed.

The aim of this study was to collect, quantify, compare, and evaluate the risk factors and identify the possible reasons for ASF introduction into different pig production farms (family farms A, B, backyards, semi and free-range) through tracking the data between years 2020 and 2022 for two districts where extensive pig production is the predominant type of pig rearing.

## 2. Materials and Methods

**Figure 1:**
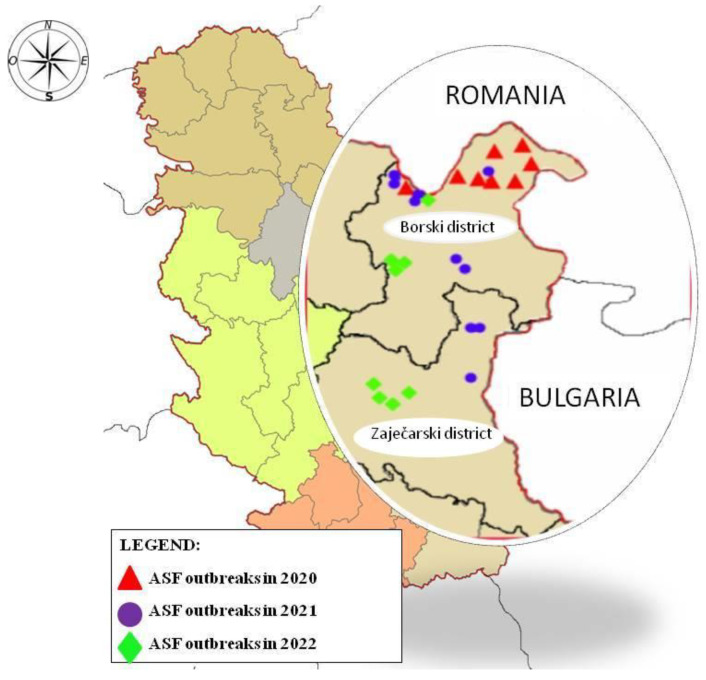
ASF outbreaks in different years in Borski district (northern) and Zaječarski district (southern).

Twenty-six pig farms or holdings with confirmed ASF outbreaks were analyzed in this study in two districts in eastern Serbia: Borski district and Zaječarski district, where 8 outbreaks were recorded in 2020 and 9 outbreaks were recorded in both 2021 and 2022 (Figure 1). After an outbreak confirmation, epidemiological investigation was performed via veterinary inspection by local epidemiologists in the presence of the farm’s owner. Collected epidemiological data were divided into 21 main categories and additional subcategories that were considered present or absent risk factors/indicators for introduction of ASF to the farm:

1. Farm location: Borski district or Zaječarski district;
2. Type of settlement: city, village, hamlet, or wood;
3. Type of holding: family farms types A and B, backyard, semi-free range, or free range;
4. Distance to hunting ground: not nearby, close/around, or in hunting ground;
5. Present pig category: boars, pregnant sows, sows, gilts, suckling piglets, weaners, and/or fatteners;
6. Diseased pig category;
7. Dead pig category;
8. Animal movement: no or yes (inside local settlement and/or inside local district and/or to other districts);
9. Home slaughtering: no or yes;
10. Fencing around the farm/yard: fenced, semi-fenced, or non-fenced;
11. Noted domestic pig–wild boar contact: no, yes, or sometimes;
12. Involvement of the farm owner in hunting activities: no, yes, or sometimes;
13. Swill feeding: no, yes, or sometimes;
14. Natural mating: no, yes, or sometimes;
15. Feed with grains from local fields; no, yes, or sometimes;
16. Feed with mowed green grass: no, yes, or sometimes;
17. Other domestic animals in the yard; no or yes (which species and how many);
18. Owner’s general agricultural activities: agriculture and/or animal husbandry and/or field work and/or work in the wood;
19. Entrance of visitors: no or yes;
20. Entrance of vehicles into the farm site premises/backyard: no or yes (agricultural vehicles and/or animal transport vehicles and/or other);
21. Reasons for keeping pigs: personal consumption and/or piglet production and/or fattener production and/or gilt production and/or nature mating.

### Statistical Analyses

Most of our data were categorical. Thus, most of our could take on a limited number of possible values, assigning each farm to a particular group (nominal category) on the basis of a given qualitative property, such as type of holding (i.e., family, backyard, semi-free range, or free range farm) or reason for keeping pigs (i.e., personal consumption, piglet production, fattener production, gilt production, or nature mating). Hence, we used contingency tables (crosstabs) to summarize the relationship between different pairs of categorical variables through observing frequencies for combinations of values for every pair of variables. The contingency tables allowed us to represent and study possible associations/dependencies between pairs of variables. In this study, relying on the relatively small sample of 26 farms, Fisher’s exact test was used. In contingency tables of size 2 × 2, the null hypothesis was assessed (i.e., the *p*-values calculated) using the hypergeometric distribution, while in contingency tables larger than 2 × 2, the *p*-values were calculated via Monte Carlo simulation (with 2000 replicates). We concluded that there was a statistically significant association between two categorical variables if the calculated *p*-value was smaller than the chosen level of significance (0.05). All the statistical analyses were performed using the program R 4.1.0 [24].

Furthermore, in the set of all observed variables, we wanted to identify important ASF transmission indicators, as we call those variables (together with specific values critical for ASF transmission) that most increase the probability of ASF transmission. Specific values of variables were considered as critical for ASF transmission according to the literature and experience (Table 1). For each variable, each farm was assigned either to the ASF transmission critical group or non-critical group, according to the farm’s qualitative property. We identified important ASF transmission indicators as those variables (together with the values critical for ASF transmission) for which a large proportion of the observed farms (at least 2/3) was assigned to the ASF transmission critical group.

## 3. Results

### 3.1. Summay of Risk Factors

In January and February 2020, all the noted infections were limited to the northern area of Borski district near the Romanian border. In the following two years, ASF spread southward into Zaječar district (Figure 1). All the farms were extensive or small scale; the largest farm had 73 pigs (5 boars, 16 pregnant sows, 13 gilts, and 39 suckling piglets; 1 boar, 3 pregnant sow, and 1 gilt died from ASF) at the time of completing the questionnaire, while some of the farms only bred one pig for personal consumption. All farms, except one, had other domestic animal species present on the premises. Only one farm from 2020 transported live pigs outside the district, while all other farms that operated inside the district only had animals for personal consumption. Home slaughtering was used in 73.05% of cases (19 of 26 farms), while the other farms used commercial slaughtering. In 76.93% of cases (20 of 26 farms), natural mating was used when breeding their sows, while the other farms either used artificial insemination of their sows or did not rear pigs for breeding purposes. Farmers either confirmed close contact between their pigs and wild boars or had seen wild boars near the premises in 34.62% of cases (9 of 26 farmers). Hunting activity and swill feeding was a part of 8 farmers’ routines (30.77%). All farms, except one (96.15%), used locally produced fresh mowed grass or grains from local fields to feed their livestock. The summary of the total number of appearances of each risk factor in presented in Table 2 and the complete year-by-year summary of noted and evaluated risk factors is presented in Appendix A (Table A1, Table A2 and Table A3).

The identification of critical ASF transmission indicators was based on values of variables that were considered critical for ASF transmission. These values, which were determined with help of the findings and experiences of previous studies [9,13,25,26], are listed in Table 1. For each observed variable, we counted the number of farms that were, due to provided values critical for ASF transmission, assigned to the ASF transmission critical group. Table 2 summarizes this counting in the sample of all 26 farms. A variable with at least 2/3 of the observed farms assigned to its ASF transmission critical group is identified as an important ASF transmission indicator. Hence, we found nine important ASF transmission indicators: “type of holding” (25 farms), “distance to hunting ground” (18 farms), “present pig category” (20 farms), “home slaughtering” (19 farms), “natural mating” (20 farms), “feed with grains from local fields” (25 farms), “other domestic animals in the yard” (25 farms), “human activities” (all 26 farms), and “entry of vehicles” (20 farms).

We also identified how many ASF transmission critical groups are classified as individual family farms that experienced ASF transmission. The minimum number of memberships is five and the maximum number of memberships is nine out of nine. The mean number of memberships is as high as 7.6 and the median is 8.

### 3.2. Comparisson and Statistical Analysis of Risk Factors

Using Fisher’s exact test on the contingency tables of pairs of observed categorical variables, we found several statistically significant associations/dependencies (Table 3).

All pairs in the group of four variables “type of holding”, ”farm/yard fencing”, ”domestic pig–wild boar contact”, and “hunting activity” are significantly related (*p* < 0.05). Thus, the arrangement of frequencies of all possible combinations of values of any two variables in this group of four in rows and columns (contingency table) is such that we may treat them as dependent variables. In particular, hunting activity of pig holders, holding pigs in backyards, fenced yards, and no domestic pig–wild boar contact are observed on the same farms. Moreover, free-range pig farming led to observed domestic pig–wild boar contact on all our farms. Another two dependent (*p* = 0.02799) variables that are worth mentioning are “movement” and “reason for keeping pigs”, where production of piglets, fatteners, and gilts, as well as natural mating, all led to (mostly local) movement. While the final dependency was expected, the exclusive dependencies between ”home slaughtering” and “feed with grains from local fields” (*p* = 0.04748), as well as between “swill feeding” and “feed with mowed green mass” (*p* = 0.01199), but not between “home slaughtering” and “swill feeding” or “feeding with mowed green mass”, are more surprising.

## 4. Discussion

In the present study, the impact of specific risk factors on ASF transmission in different traditional extensive farms in Serbia was analyzed and compared. Since the first case was recorded in 2019, outbreaks of ASF were detected every year in Serbia, and the disease became endemic in some regions of Serbia in both wild boars and extensive domestic pig herds [12]. It is generally accepted that to prevent the spread of ASF back and forth from wild boar to domestic pig populations, external biosecurity measures must be implemented and controlled in the pig production sector. An important characteristic of backyards and various types of family (small-scale) farms in this Serbia is their inadequate biosecurity. The common characteristics of these types of farms are low numbers of breeding animals and low piglet and fattener production, non-professional farm management, traditional home slaughtering, and production of various homemade meat products (e.g., sausage, ham, and bacon). In addition, natural mating with breeding boars is widespread. Although swill feeding is officially prohibited in Serbia, it is quite difficult to control it in remote rural areas [14,15,27].

Our study did not aim to reveal the specific entry point of ASF virus into the different types of extensive pig farms; rather, it aimed to provide a retrospective, broad-based analysis of all risk factors potentially involved (to a greater or lesser extent) in ASF transmission. The study aimed to uncover the regionally specific situation regarding extensive family pig farming, backyards, and semi-free-range or free-range farming around villages. Regarding intensive commercial pig production in these districts, an ASF outbreak was reported in 2021 at a large pig farm in Zaječar district [16], which was the only commercial pig farm in this region. This result shows that biosecurity measures applied in intensive pig farms do not always work in practice and are highly influenced by ASF contamination of the adjacent area and the habits, traditions, and mentality of people involved in pig production and in hunting. The analyses revealed critical risk factors that affected ASF transmission in various extensive farms in Borski and Zaječarski districts, and these factors are probably the most important to consider in preventing further spread. “Type of settlement” and “type of holding” were shown as two of the most important factors; however, these factors are rather hard to be affected by any applicable measures, unless government shuts down these types of pig operations in the high-risk areas. The factors identified as the most important in this study that can be affected by implementing biosecurity measures and changing established human behavior are ”farm/yard fencing”, which is related to prevention of direct ”domestic pig–wild boar contact” and the “hunting activity” of pig holders (Table 3). Results from the survey also indicate that extensive pig units in the study are concerning in terms of the numbers of observed risk factors for ”home slaughtering”, “natural mating”, location in or at a close “distance to hunting grounds” (i.e., exposure to domestic pig–wild boar contact (even if not observed), having “other domestic animals” in the yard and different “vehicles” entering the premises, and yards with no applicable preventive measures (cleaning and disinfection of transport or different agricultural vehicles). Indeed, this is not surprising given the characteristics of the extensive pig units analyzed in the region. The statistical analysis revealed some correlations that may be logical/expected, as well as some that may be less expected and could be given more consideration in the future to prevent transmission of ASF from wild boar populations and/or contaminated habitats to domestic swine populations. At this point, it is important to emphasize that this is a numerical retrospective analysis and the actual time of introduction in any given situation was not determined. Therefore, all risk factors and biosecurity measures should be considered and implemented to the extent possible. Firstly, the risk factors need to be identified and defined, possibly in a similar way as Andraud et al. did during the ASF outbreak in Romania in 2021 [22], or in Slovenia, where assessment was performed even though ASF was not detected [28]. Some of these factors are significant but cannot be affected or prevented due to geography of the terrain [29,30], farm location, farm size [26], the proximity of the woods and fields, the type of vegetation [31], and the wild boar population present in the surrounding area. However, there is human-related tradition, i.e., customs in extensive pig production, that can be addressed to change the current epidemiological situation. The fencing of the farm area or pasture, when pigs are allowed to be kept outside, is clearly not implemented by farmers; 57.69% (15 out of 26) of farmers have only partial fencing or no fencing at all. This problem is a risk factor that could be avoided through a targeted awareness campaign and education of farmers and pig owners, as well as possibly through investment funded by the relevant authorities [32]. Placing fences around the site in remote areas can be challenging. Bosch et al. proposed an approach that not only fences yards containing domestic pigs, but also establishes wild boar culling zones (white zones). Through mapping high-risk areas for ASF occurrence in wild boar based on the criteria identified, it may be possible to identify landscape corridors of high and low disease risk [33]. It is in our interest to point out to pig owners that it would be better to stop hunting, or at least to classify hunting as very risky, and that the owner should not come into contact with domestic pigs for 48–72 h after hunting; however, in the EFSA study of Estonian cases, no association between hunting activities and ASF transmission was found [13], though that is only true in the light of different customs and biosecurity measures used during and after hunting activity, as well as many other variables, which is also stated by Pepin et al. in [34]. The presence of wild boar population poses an extremely high risk [9,34], though not all studies came to the same conclusions; in Estonia [26], Poland [25], and Czech Republic [29], the mere presence of wild boar did not extend the possibility for transmission to domestic pigs, though it was supported by human activity. Home slaughtering is often conducted in private slaughter rooms on the premises or directly in the backyard in the open air; especially in the latter manner, ASF virus can spread and persist in the soil and may be further transmitted through rain and human activity [15,35]. As for natural mating, it not only supports direct contact between different categories of pigs, but in cases of extensive pig farming, it often requires the movement of animals between farms and should, therefore, not be carried out in areas with a high risk of ASF transmission [35,36]. Currently, there are no detailed reports on the role of other species of domestic vertebrates as mechanical vectors for ASF transmission. Other animals could theoretically carry the virus on their exterior, or in the case of keeping several different species of livestock, increased spread could be related to increased human activity. Movement of vehicles is a known critical factor in ASF transmission over short distances and long distances, as in the case of the Czech Republic [29] and Belgium [37]. Another critical factor that needs to be addressed is the feeding of home-produced feed made from the grains produced in the farm’s own fields. This practice is common and is intended to reduce the additional cost of purchasing competing swine feed; however, the government could limit transmission through helping farms purchase locally produced feed and use it for other purposes not related to feeding domestic pigs. It is important that pigs are not fed swill, which is already prohibited. However, there are no practical measures to monitor and penalize farmers with such small operations; the results of the 2021 study conducted by Mauroy et al. ranked swill feeding as the fifth greatest risk for ASF transmission out of twenty-five risks assessed [38]. From the results of this study, it appears that hunting and swill feeding decreased over the years; in 2022, only one of the farmers surveyed either hunted or fed swill, though with such a small sample, this could be coincidence. However, if ASF outbreaks are to be prevented in the future, there needs to be a significant change in the attitude of this type of farmer, as well as some government action (e.g., awareness campaigns in large-scale areas and financial support for external biosecurity measures), which should be practical to implement [39].

After first ASF outbreak in the country, the Serbian authorities are working closely with international organizations, such as the World Organization for Animal Health (WOAH) and the Food and Agriculture Organization (FAO), to manage the outbreak and prevent its spread. They implemented measures such as movement restrictions, disinfection protocols, and extensive active and passive surveillance activities to detect and control the ASF; however, it might be necessary to tighten the restrictions further with different kind of measures. It is important for all stakeholders in the pork industry, including farmers, processors, and consumers, to remain vigilant and take appropriate precautions and biosecurity measures to prevent the future spread of ASF. It should be stressed that humans are frequently recognized as the decisive and nature-independent factor that often unintentionally contributes to the spread of ASF and connects two distinct populations: domestic pigs and wild boars.

## 5. Conclusions

Different types of extensive pig farms, in combination with the customs, traditions, and mindsets of individuals involved in pig production, pose the biggest threat for the spread of ASF to domestic pigs in Serbia. According to the results, which indicated the most important risk factors for ASF transmission to these types of farms, it can be concluded that, although all variables cannot be impacted, some may be improved. Farmers should provide shelter or fencing around the farm/yard that would prevent the direct domestic pig–wild boar contact, avoid hunting activity or strictly separate it from farming activities, limit human activity to a necessary minimum (visitors, transport vehicles), and stop feeding domestic pigs with swill or any unprocessed fresh feed that are more likely to contain infective ASF virus.

## Figures and Tables

**Table 1:** ASF transmission critical and non-critical values by variable.

Variable	Factors Critical for ASF Transmission	Factors Non-Critical for ASF Transmission
Type of holding	Backyard, semi-free range, free range	Small holding
Distance to hunting ground	close/around infected nearby hunting ground, in infected hunting ground	Not nearby
Present pig category	Boars, sows, pregnant sows	Gilts, suckling piglets, weaned piglets, fatteners
Movement	Local village, local district, other district	No
Home–slaughtering	Yes	No
Farm/yard	Semi-fenced, non-fenced	Fenced
Domestic pig–wild boar contact	Yes, sometimes	No
Hunting activity	Yes, sometimes	No
Swill feeding	Yes, sometimes	No
Natural mating	Yes, sometimes	No
Feed with grains from local fields	Yes, sometimes	No
Feed with mowed green mass	Yes, sometimes	No
Other domestic animals in the yard	Yes (cattle, goat, horse, dog, poultry, sheep, cat)	No
Human activities	Agriculture, animal husbandry, field work, other	No
Entry of other people	Yes	No
Entry of vehicles	Agriculatural vehicles, animal transport vehicles	No
Reason(s) for keeping pigs	Exclusively personal consumption	Piglets production, fatteners production, gilts production, nature mating

**Table 2:** Counted total amount of ASF transmission risk factors by year (numerical only).

	2020 (N = 8)	2021 (N = 9)	2022 (N = 9)	All (N = 26)
Type of holding	8	9	8	25
Distance to hunting ground	7	4	7	18
Present pig category	5	7	8	20
Movement	5	3	5	13
Home slaughtering	6	7	6	19
Farm/yard fencing	6	3	6	15
Domestic pig–wild boar contact	6	3	4	13
Hunting activity	2	5	1	8
Swill feeding	4	3	1	8
Natural mating	7	5	8	20
Feed with grains from local fields	7	9	9	25
Feed with mowed green mass	5	3	3	11
Other domestic animals in the yard	8	9	8	25
Human activities	8	9	9	26
Entry of other people	3	3	3	9
Entry of vehincles	3	9	9	21
Reason for keeping pigs	3	7	4	14

N—number of farms per year.

**Table 3:** Correlation between parameters and *p*-value confirming statistical significance of their relationship.

1st Parameter	2nd Parameter	*p*-Value
Type of settlement	Type of holding	0.0004998
	Distance to hunting ground	0.002999
	Farm/yard fencing	0.001999
	Domestic pig–wild boar contact	0.0004998
	Entry of other people	0.02199
Type of holding	Farm/yard fencing	0.0004998
	Domestic pig–wild boar contact	0.0004998
	Hunting activity	0.03248
Dead category	Feed with mowed green mass	0.04648
Movement of animals	Reason for keeping pigs	0.02799
Home slaughtering	Feed with grains from local fields	0.04748
Farm/yard fencing	Domestic pig–wild boar contact	0.0004998
	Hunting activity	0.001499
Domestic pig–wild boar contact	Hunting activity	0.001499
Swill feeding	Feed with mowed green mass	0.01199

## Data Availability

All original data in this study are available from the authors of the study upon request. Publicly available portals are listed in the References section.

## Appendix A

**Table A1:** List of noted epidemiological risk factors from ASF-positive farms in 2020.

		F1	F2	F3	F4	F5	F6	F7	F8
Location	Borski	+	+	+	+	+	+	+	+
	Zaječarski								
Settlement	Suburb								+
	Village			+	+	+			
	Hamlet	+	+				+	+	
	Woods								
Holding	Smallholding								
	Backyard			+	+	+	+	+	+
	Semi-free range	+	+		+	+	+	+	
	Free-range	+	+						
Distance to hunting ground	Not nearby								+
	Close	+	+	+	+	+	+	+	
	In infected hunting ground								
Present pig category	Boars				1			1	1
	Pregnant sows								
	Sows				18	1	1	1	
	Gilts		1						7
	Suckling piglets				11		6		
	Weaned piglets		8					6	
	Fatteners	1		1	8				
Diseased pig category		none *	1S	1Fat	1S	none *	1S	1B, 6W	1B, 7G
Dead pig category		1S	1S	none	1S	1S, 1W	1S	1B	1B
Animal movement	No			+		+	+		
	Local village	+						+	
	Local district		+		+				+
	Other district				+				
Home slaughtering	No	+							+
	Yes		+	+	+	+	+	+	
Fencing	Yes			+					+
	Semi-fenced				+	+	+		
	None	+	+					+	
Domestic pig–wild boar contact	No			+					+
	Yes	+	+		+	+	+	+	
	Sometimes								
Hunting activities	No	+	+		+	+	+	+	
	Yes			+					+
	Sometimes								
Swill feeding	No		+	+	+				+
	Yes	+				+	+	+	
	Sometimes								
Natural mating	No			+					
	Yes	+	+		+	+	+	+	+
	Sometimes								
Feed with grains from local fields	No								+
	Yes	+	+	+	+	+	+	+	
	Sometimes								
Feed with mowed green grass	No			+	+				+
	Yes							+	
	Sometimes	+	+			+	+		
Other domestic animals	No								
	Yes	+	+	+	+	+	+	+	+
Owner’s agricultural activities	Agriculture	+	+	+	+	+	+	+	
	Animal husbandry				+				
	Field work	+	+	+	+		+		
	Other							+	+
Visitors	No	+	+			+	+	+	
	Yes			+	+				+
Vehicles	Agriculatural vehicles	+	+		+				
	Animal transport vehicles				+				
	None	+	+	+		+	+	+	+
Reasons for keeping pigs	Personal consumption	+	+	+	+	+	+	+	+
	Piglet production		+		+			+	+
	Fattener production	+							
	Gilt production								
	Nature mating								

* all pigs on farm died from ASF. F—farm; S—sow; Fat—fattener; W—weaner; G—gilt; B—boar; + symbol marks the answer of the owner was positive/affirmative.

**Table A2:** List of noted epidemiological risk factors from ASF-positive farms in 2021.

		F1	F2	F3	F4	F5	F6	F7	F8	F9
Location	Borski	+	+	+	+	+	+			
	Zaječarski							+	+	+
Settlement	Suburb									
	Village	+	+	+	+	+	+	+	+	
	Hamlet									+
	Woods									
Holding	Smallholding									
	Backyard	+	+	+	+	+	+	+	+	
	Semi-free range									+
	Free-range									
Distance to hunting ground	Not nearby	+				+	+	+	+	
	Close		+	+	+					+
	In infected hunting ground									
Present pig category	Boars					1				1
	Pregnant sows	+				1				
	Sows		2				2	2	3	2
	Gilts					2	2			4
	Suckling piglets						8		5	
	Weaned piglets									
	Fatteners		3			2	3		2	7
Diseased pig category		1F	1F	None *	None *	All	All	1S	2S	1G
Dead pig category		1F	1F	1B, 1F	1S	None	1S	None	1S	1G
Animal movement	No	+	+	+	+		+	+		
	Local village					+			+	
	Local district									+
	Other district									
Home-slaughter	No	+			+					
	Yes		+	+		+	+	+	+	+
Fencing	Yes	+	+	+	+			+	+	
	Semi-fenced					+	+			+
	None									
Domestic pig–wild boar contact	No	+	+	+	+			+	+	
	Yes									
	Sometimes					+	+			+
Hunting activities	No	+	+			+				+
	Yes			+	+			+	+	
	Sometimes						+			
Swill feeding	No	+				+	+	+	+	+
	Yes		+	+	+					
	Sometimes									
Natural mating	No	+		+		+		+		
	Yes		+		+		+		+	+
	Sometimes									
Feed with grains from local fields	No									
	Yes		+			+	+	+	+	+
	Sometimes	+		+	+					
Feed with mowed green grass	No				+	+	+	+	+	+
	Yes	+		+						
	Sometimes		+							
Other domestic animals	No									
	Yes	+	+	+	+	+	+	+	+	+
Owner’s agricultural activities	Agriculture				+	+	+	+	+	+
	Animal husbandry						+			
	Field work	+	+	+		+		+	+	+
	Other		+		+					
Visitors	No	+		+		+		+	+	+
	Yes		+		+		+			
Vehicles	Agriculatural vehicles		+	+	+	+	+	+	+	+
	Animal transport vehicles		+							
	None	+								
Reasons for keeping pigs	Personal consumption	+	+	+	+	+	+	+	+	+
	Piglets production								+	+
	Fatteners production									
	Gilts production									
	Nature mating									

* all pigs on farm died from ASF. F—farm; S—sow; G—gilt; B—boar; + symbol marks the answer of the owner was positive/affirmative.

**Table A3:** List of noted epidemiological risk factors from ASF-positive farms in 2022.

		F1	F2	F3	F4	F5	F6	F7	F8	F9
Location	Borski		+				+	+	+	+
	Zaječarski	+		+	+	+				
Settlement	Suburb									
	Village	+	+	+	+	+				+
	Hamlet						+	+	+	
	Woods						+	+	+	
Holding	Smallholding					+				
	Backyard	+	+	+	+					
	Semi-free range	+					+	+	+	+
	Free-range						+	+	+	
Distance to hunting ground	Not nearby	+								+
	Close		+	+		+				
	In infected hunting ground				+		+	+	+	
Present pig category	Boars	3			1	5	2	1	1	1
	Pregnant sows	32				16	5	1		2
	Sows	4	4				1			2
	Gilts	1				13		1		
	Suckling piglets					39	10			
	Weaned piglets						6		1	
	Fatteners			3						
Diseased pig category		None *	All	All	1B	1B, 6PS, 1G	2S	1PS	All	All
Dead pig category		2S, 1G	1PS	2S	None	1B, 3PS, 1G	1B, 1PS	None	1B, 1S, 1SP	None
Animal movement	No				+			+	+	+
	Local village	+	+	+		+	+			
	Local district	+	+			+				
	Other district									
Home-slaughter	No				+			+		+
	Yes	+	+	+		+	+		+	
Fencing	Yes		+	+	+					
	Semi-fenced	+				+	+	+	+	+
	None									
Domestic pig–wild boar contact	No		+	+	+	+				+
	Yes	+					+	+	+	
	Sometimes									
Hunting activities	No	+	+		+	+	+	+	+	+
	Yes			+						
	Sometimes									
Swill feeding	No	+	+	+	+	+	+	+	+	
	Yes									+
	Sometimes									
Natural mating	No				+					
	Yes	+	+	+		+	+	+	+	+
	Sometimes									
Feed with grains from local fields	No									
	Yes	+	+	+	+	+	+	+	+	+
	Sometimes									
Feed with mowed green grass	No	+	+				+	+	+	
	Yes				+	+				
	Sometimes			+						
Other domestic animals	No	+								
	Yes		+	+	+	+	+	+	+	+
Owner’s agricultural activities	Agriculture	+	+	+	+	+	+	+	+	+
	Animal husbandry					+	+			+
	Field work	+	+	+	+	+	+	+	+	+
	Other									
Visitors	No			+	+	+	+	+	+	
	Yes	+	+							+
Vehicles	Agriculatural vehicles	+	+	+	+	+	+	+	+	+
	Animal transport vehicles	+	+			+				+
	None									
Reasons for keeping pigs	Personal consumption	+	+	+	+	+	+	+	+	+
	Piglets production	+	+			+	+			
	Fatteners production			+		+				
	Gilts production									
	Nature mating					+				

* all pigs on farm died from ASF. F—farm; S—sow; G—gilt; B—boar; PS—pregnant sow; SP—suckling piglet; + symbol marks the answer of the owner was positive/affirmative.
